# An efficient system for homology-dependent targeted gene integration in medaka (*Oryzias latipes*)

**DOI:** 10.1186/s40851-017-0071-x

**Published:** 2017-07-06

**Authors:** Yu Murakami, Satoshi Ansai, Akari Yonemura, Masato Kinoshita

**Affiliations:** 1 Division of Applied Bioscience, Graduate School of Agriculture, Kyoto University, Kitashirakawa-Oiwake-cho, Sakyo-ku, Kyoto, 606-8502 Japan; 2 Present address: Division of Ecological Genetics, Department of Population Genetics, National Institute of Genetics, Yata 1111, Mishima, Shizuoka, 411-8540 Japan

**Keywords:** Bait sequence, CRISPR/Cas9, Homology-directed repair, Targeted gene integration, Medaka

## Abstract

**Background:**

The CRISPR/Cas system is a powerful genome editing tool that enables targeted genome modifications in various organisms. In medaka (*Oryzias latipes*), targeted mutagenesis with small insertions and deletions using this system have become a robust technique and are now widely used. However, to date there have been only a small number of reports on targeted gene integration using this system. We thus sought in the present study to identify factors that enhance the efficiency of targeted gene integration events in medaka.

**Results:**

We show that longer homology arms (ca. 500 bp) and linearization of circular donor plasmids by cleavage with bait sequences enhances the efficiency of targeted integration of plasmids in embryos. A new bait sequence, BaitD, which we designed and selected by in silico screening, achieved the highest efficiency of the targeted gene integration in vivo. Using this system, donor plasmids integrated precisely at target sites and were efficiently transmitted to progeny. We also report that the genotype of F_2_ siblings, obtained by mating of individuals harboring two different colors of fluorescent protein genes (e.g. GFP and RFP) in the same locus, can be easily and rapidly determined non-invasively by visual observations alone.

**Conclusion:**

We report that the efficiency of targeted gene integration can be enhanced by using donor vectors with longer homologous arms and linearization using a highly active bait system in medaka. These findings may contribute to the establishment of more efficient systems for targeted gene integration in medaka and other fish species.

**Electronic supplementary material:**

The online version of this article (doi:10.1186/s40851-017-0071-x) contains supplementary material, which is available to authorized users.

## Background

Medaka (*Oryzias latipes*) is a small freshwater teleost species, which serves as an excellent vertebrate model for genetics due to it ease of breeding and unique genetic resources, such as spontaneous mutant collections, highly polymorphic inbred strains, and related species that exhibit a number of unique features [1, 2]. In medaka, a large number of transgenic strains is available for molecular genetic analysis [2, 3]. However, the development of techniques for targeted manipulation of endogenous genes, such as genetic tagging by reporter genes and site-specific introduction of single nucleotide polymorphisms, has been hindered by lack of established protocols for gene targeting in embryonic stem cells in this species.

Genome editing using targetable nucleases, including systems utilizing clustered regularly interspaced short palindromic repeats (CRISPR) and CRISPR-associated (Cas), transcription activator-like effector nucleases (TALENs), and zinc-finger nucleases (ZFNs), have been established as a powerful methods for reverse genetics in a wide range of organisms [4]. These nucleases can induce DNA double-strand-breaks (DSBs) at any genomic target locus, which allows for various types of targeted genome modifications via DNA DSB repair systems, such as targeted gene disruptions by small insertions and deletions (indels) via non-homologous end-joining (NHEJ) and targeted gene integration by homology-directed repair (HDR) [5]. We previously reported targeted gene disruptions mediated by NHEJ, thus establishing efficient methods for targeted mutagenesis using targetable nuclease systems in medaka [6–8]. However, although targeted gene integration mediated by HDR is desirable for more precise and complex genome manipulations, there have been only a few reports on the HDR-mediated gene integration in medaka [9]. More detailed knowledge is thus needed to establish efficient protocols for this technology.

The length of homologous sequences is known to play an important role in determining the pathways used for the repair of DNA DSBs [10]. Relatively long homology arms (0.5–1 kb) can induce homologous recombination (HR) and have been commonly used for targeted gene integration by genome editing [5]. Short homology arms (2–25 bp) in contrast can induce microhomology-mediated end joining (MMEJ), recently identified as a DSB repair pathway in a highly efficient gene knockin method for genome editing [11, 12]. Recent studies have reported that both of these pathways can mediate targeted gene integration in zebrafish embryos [13–15]. However, no studies have directly compared the integration efficiencies mediated by these two pathways in fish embryos, and thus the effects of length of homology arms on integration efficiency have remained unclear.

Previous studies have also demonstrated that simultaneous cleavage of a circular donor plasmid and the targeted genomic locus by targetable nucleases can enhance HDR-mediated gene integration efficiency in zebrafish and sea urchin embryos [13, 16]. For efficient methods of the targeted gene integration by CRISPR/Cas system, guide RNAs (gRNAs) and their targets, known as “bait” sequences, have been designed for the cleavage of circular donor plasmids. Gbait is a bait sequence designed on the coding sequence of *EGFP* gene, and has been used in several zebrafish studies due to its high genome-editing activity and lower frequency of off-target effects. In addition, PITCh gRNAs were designed for targeted integration by the PITCh system, and has also minimized off-target effects in various mammalian genomes [12]. However, there has been no comparative studies on the effect of these sequences on the efficiency of targeted gene integration in vivo.

In the present study, we sought to establish an efficient system for targeted gene integration in medaka. First, we examined the effects of the length of homology arms on integration efficiencies at a genomic locus in medaka embryos. Next, we developed novel bait sequences with fewer off-target effects in fish genomes and validated their effect on integration efficiency in comparison with the previously reported bait sequences, Gbait and PITCh gRNAs. Lastly, we demonstrated that gene knockin strains harboring different fluorescent protein genes at the target locus can be generated by a method established in this study and that these strains may be helpful in maintaining mutant strains without PCR genotyping.

## Methods

### Ethics statement

This study was conducted in compliance with the Regulation for Animal Experiments in Kyoto University. Fish handling and sampling methods were approved by Kyoto University (No.26–71). All efforts were made to minimize suffering.

### Fish

A cab (closed colony) of medaka was used in this study. The fish were kept under a 14/10-h light/dark cycle at 26 °C.

### Design of bait sequences

Candidate bait sequences that are disrupted by corresponding single guide RNAs (sgRNAs) were designed following published data sets from high-throughput screening of sgRNA activity in mammalian cells [17]. The top 40 sgRNAs with the highest gene disrupting activity (20 from the “non ribo efficient sgRNA” data set and 20 from the “mESC efficient sgRNA” data set) were nominated as candidates. These candidates were screened by following two criteria using an offline version of Cas-OFFinder [18] with a genome database of 12 teleost fish species (Additional file 1: Table S1). For each candidate, we calculated the total number of potential off-target sequences with ~3 bp mismatches in 18 bp of target sequence and PAM (NGG or NAG) sequence (in total 21 bp) in the 12 fish genome database. From this group, the seven candidates with the lowest numbers of total potential off-target sequences were selected. We screened the selected candidates and the previously reported bait sequences (Gbait [19] and PITCh-gRNAs [12]) following the second criterion: genomic sequences with ~2 bp mismatches in the 18-bp target sequence for each sgRNA [8, 20]. After calculating the total number of off-target sequences in the12 fish genome database, the eight bait sequences with the lowest number were selected for use in this study.

### Construction of donor plasmids

#### Backbone fragment 1

Backbone fragments containing a pUC replication origin and an ampicillin resistance gene were amplified from a plasmid pPBIS19-*mgfc*:Tag*BFP*-8x*HSE*:*Cre* [21] by PCR using a primer pair (pUCoriFW-SpeI and pUCoriRV-XhoI) (Additional file 2: Table S4) and digested with XhoI and SpeI.

#### Backbone fragment 2

Backbone fragments containing each bait sequence, a pUC replication origin, and an ampicillin resistance gene were amplified from a plasmid pPBIS19-*mgfc*:Tag*BFP*-8x*HSE*:*Cre* [21] by PCR using a primer pair (SpeI-Bait-FW and XhoI-Bait-RV) with each bait sequence and a restriction site (XhoI or SpeI) (Additional file 2: Table S4) and then digested with XhoI and SpeI.

GFP cassette: To avoid *EGFP* gene disruption induced by Gbait, we used *monomeric Azami-Green* (*mAG*) gene as a reporter gene expressing green fluorescence protein (GFP). A GFP reporter cassette, a *mAG* gene with a N-terminal linker and a SV40 polyA signal, was amplified from a plasmid phmAG1-MNLinker (Medical & Biological Laboratories, Aichi, Japan) by PCR using primers mAG-linker-BamHI and polyA-RV-EcoRI (Additional file 2: Table S4), and then digested with BamHI and EcoRI.

pBaitX-acta1_500 bp-mAG (Fig. 2a): To generate donor plasmids with ~500 bp of sequences homologous to *acta1* locus (pBaitX-acta1_500 bp-mAG), upstream (472 bp) or downstream (473 bp) genomic region at the target site of sgRNA-acta1 was PCR-amplified using primer pairs with restriction sites, acta1-5FW-XhoI/acta1-5RV-BamHI or acta1-3FW-EcoRI/acta1-3RV-SpeI (Additional file 2: Table S4) and then digested with XhoI/BamHI or EcoRI/SpeI, respectively. These two fragments were simultaneously ligated with the “Backbone fragment 2” and the “GFP cassette”.

**Fig. 1:**
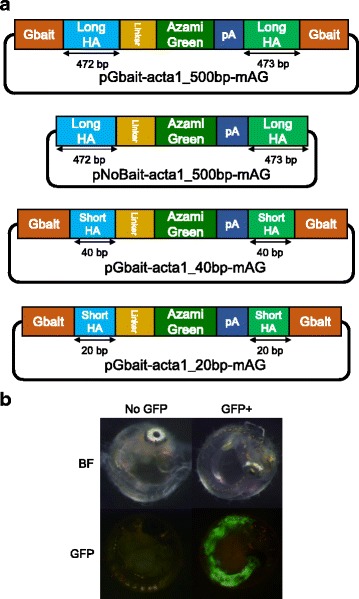
Effects of the length of homologous sequences and presence of bait sequence on the efficiencies of targeted gene integration. (a) Schematics of each donor plasmid for evaluating the effects of homologous sequences and the bait sequence. The pGbait-acta1_500 bp-mAG plasmid contains long homology arms (Long HAs) and Gbait. The pNoBait-acta1_500 bp-mAG plasmid is the plasmid that removed the Gbait from pGbait-acta1_500 bp-mAG plasmid. The pGbait-acta1_40 bp-mAG plasmid contains 40 bp short homology arms (Short HAs) and Gbait. The pGbait-acta1_20 bp-mAG plasmid contains 20 bp short homology arms (Short HAs) and Gbait. (b) Embryos at 4 days post fertilization (dpf). Embryos injected with each plasmid, Cas9 RNA (100 ng/μl) with or without Gbait-sgRNA were categorized into the following two groups; “No GFP” for embryos without green fluorescence and “GFP+” for embryos with fluorescence in the skeletal muscle, respectively. BF: bright field, GFP: GFP fluorescent image

pNoBait-acta1_500 bp-mAG (Fig. 1a): The “Backbone fragment 1” and the “GFP cassette” are ligated to generate a donor plasmid without bait sequences.

pGbait-acta1_20 bp-mAG (Fig. 1a): Single-stranded oligo DNAs of acta1-exon3-5S1 and acta1-exon3-5AS1 or acta1-exon3-3S1 and acta1-exon3-3AS1 (Additional file 2: Table S4) were annealed to generate double-stranded oligo DNAs containing upstream or downstream short homology arms (20 bp). These double-stranded oligo DNAs were ligated with the “GFP cassette” and one of the “Backbone fragment 2,” which contains the Gbait to generate the pGbait-acta1-short-mAG donor plasmid.

pGbait-acta1_40 bp-mAG (Fig. 1a): To generate a donor plasmid containing short homology arms (40 bp), a fragment containing “GFP cassette” and homology arms (40 bp) was amplified from the above plasmid, pGbait-acta1_500 bp-mAG, using primer pairs XhoI-acta1-Fw and acta1-SpeI-Rv. The fragment was digested with XhoI and SpeI, and ligated with one of the “Backbone fragment 2,” which contains the Gbait to generate the pGbait-acta1_40 bp-mAG donor plasmid.

pBaitD-gap43_500 bp-mAG (Fig. 3a): To construct a donor plasmid for targeting *gap43* locus (pBaitD-gap43_500 bp-mAG), an upstream (483 bp) or downstream (496 bp) genomic region at the target site of sgRNA-gap43 was amplified by PCR with primer pairs XhoI-GAP43-FW/GAP43-BamHI-RV or EcoRI-GAP43-FW/GAP43-SpeI-RV (Additional file 2: Table S4) and digested with XhoI/BamHI or EcoRI/SpeI, respectively. These fragments and the BamHI/EcoRI-digested GFP cassette were cloned into one of the “Backbone fragment 2” containing BaitD sequences.

pBaitD-gap43_500 bp-tdTomato (Fig. 3a): The coding sequence of the *tdTomato* gene was amplified from ptdTomato Plasmid (Clontech, California, USA) by PCR using primer pairs BamHI-tdTomato-Fw/tdTomato-XbaI-Rv (Additional file 2: Table S4). The donor plasmid, pBaitD-gap43_500 bp-tdTomato, was generated following the method also used for generating pBaitD-gap43_500 bp-mAG using the tdTomato coding sequence instead of the “GFP cassette”.

All constructed plasmids were purified by an alkaline lysis method or NucleoSpin Plasmid QuickPure kit (MACHEREY-NAGEL, Düren, Germany).

### Preparation of donor plasmids, Cas9 RNA and sgRNAs for microinjection

To eliminate residual RNase activity of extracted plasmids, donor plasmids dissolved with 50 μL of 5 mM Tris-HCl buffer (pH 8.5) were incubated with 5 μL of 10% sodium dodecyl sulfate (SDS) and 2 μL of Proteinase K (20 mg/mL) at 55 °C for 30 min, and then purified using NucleoSpin Gel and PCR Clean-up kit (MACHEREY-NAGEL) with the Buffer NTB supplied by the manufacturer.

Cas9 RNA was transcribed from pCS2 + hSpCas9 (Addgene Plasmid 51,815) [8] using the mMessage mMachine SP6 Kit (Thermo Fisher Scientific, Waltham, MA). Custom-designed sgRNAs for the genomic sequence of medaka were designed using a track for the UCSC genome browser of CRISPRscan [22]. Expression plasmids for the custom-designed sgRNAs were constructed by cloning the annealed oligonucleotides into a sgRNA expression plasmid pDR274 (Addgene Plasmid 42,250) [23], as described previously [8]. The sgRNAs were transcribed from the DraI-digested template plasmids using the Ampliscribe T7-Flash Transcription Kit (Epicentre, WI). All synthesized RNAs were purified using the RNeasy Plus Mini Kit (Qiagen, Hilden, Germany) to eliminate the template DNA without DNase treatment. Sequences of the genomic target sites and annealed oligonucleotides are listed in Additional file 2: Table S4.

### Microinjection

To evaluate the DSB inducing activity of each sgRNA at the genomic target site, an injection mixture containing 100 ng/μL of Cas9 RNA and 50 ng/μL of each sgRNA was prepared. To evaluate the efficiency of targeted gene integration, injection mixtures containing 2.5 ng/μL of each donor plasmid, 100 ng/μL of Cas9 RNA, and 50 ng/μL of sgRNA corresponding to the donor plasmid were prepared. These injection mixtures were introduced into medaka eggs at the one-cell stage, as described previously [24].

### Genomic DNA extraction

Embryos were lysed individually at 4 days post fertilization (dpf) in 25 μL of alkaline lysis buffer containing 25 mM NaOH and 0.2 mM EDTA and incubated at 95 °C for 15 min after breaking the egg envelope with forceps. Each sample was neutralized with 25 μL of 40 mM Tris-HCl (pH 8.0) and used as a genomic DNA sample.

### Heteroduplex mobility assay

Heteroduplex mobility assay (HMA) was performed to investigate the DSB inducing activity of each sgRNA on the genomic target site, as described previously [7]. Briefly, a genomic region containing the target sequence of sgRNAs-acta1 or sgRNA-gap43 was amplified by PCR using KOD-FX DNA polymerase (Toyobo, Osaka, Japan) with primers acta1-HMA-FW and acta1-HMA-RV or GAP43-for-HMA-FW and GAP43-for-HMA-RV (Additional file 2: Table S4), respectively. The resulting amplicons were analyzed using a microchip electrophoresis system (MCE-202 MultiNA; Shimadzu, Kyoto, Japan) with DNA-500 reagent kit.

### Microscopic observation

Embryos and larvae injected with the donor plasmids were observed using a fluorescence stereomicroscope MZFLIII (Leica Microsystems, Wetzlar, Germany) with a GFP2 filter set (for GFP) and a DsRed filter set (for RFP). Microscopic images were captured using a digital color-cooled charge-coupled camera and the VB-7010 image control system (Keyence, Osaka, Japan).

### Sequence analysis

To evaluate the precise targeted integration, the DNA sequence around the integration site was investigated. The junction regions of the target site on the host genome and introduced gene were amplified by PCR using the following primer pairs: acta1-for-Seq-Fw and mAG-Rv, mAG-Fw and acta1-for-Seq-Rv, or GAP43-for-Seq-FW and GAP43-for Seq-RV (Additional file 2: Table S4), and KOD -plus- Neo DNA polymerase (Toyobo). The PCR conditions were as follows: one cycle at 94 °C for 2 min, followed by 35 cycles of 98 °C for 10 s, 58 °C for 30 s, and 68 °C for 1 min. The resulting PCR products were subjected to electrophoresis with a 1% agarose gel. PCR fragments predicted to contain the introduced gene were excised from the gel and purified using NucleoSpin Gel and PCR Clean-up (MACHEREY-NAGEL). The purified fragments were sequenced using the primers acta1-for-Seq-Fw and acta1-for-Seq-Rv (for *acta1*) or GAP43-for-Seq-FW and GAP43-for-Seq-RV (for *gap43*) (Additional file 2: Table S4).

## Results

### Selection of sgRNA targeting to the skeletal muscle-specific actin gene in medaka genome

Selection of a genomic target locus expressed widely in the early-stage embryo and an sgRNA possessing high genome-editing activity at that locus is necessary for the accurate and rapid evaluation of the gene knockin efficiency of each donor plasmid. A skeletal muscle-specific actin gene (*acta1*; Ensembl gene number ENSORLG00000010881) was selected as the target locus, as detection of site-specific integration by observing the green fluorescence in the skeletal muscles is simple [25, 26].

To obtain an sgRNA targeting the *acta1* gene with high DSB-inducing activity, we designed two sgRNAs targeting the third exon of the gene without potential off-target sites in the medaka genome (Additional file 3: Figure S1). Each sgRNA was injected with a Cas9 RNA into fertilized medaka eggs and its genome-editing activity was evaluated by HMA. More multiple banding patterns were observed in embryos with sgRNA-acta1 #1 than with sgRNA-acta1 #2, suggesting that sgRNA-acta1 #1 has higher DSB-inducing activity than sgRNA-acta1 #2 (Additional file 3: Figure S1). Thus, we used sgRNA-acta1 #1 for targeted genome cleavage in the following experiments.

### Effect of length of homologous sequences and existence of the bait sequence on targeted gene integration events

To assess the effects of lengths of homologous sequences that are located on both ends of the inserted gene fragment in the donor plasmid, the targeted gene integrating efficiency into the third exon of *acta1* gene was evaluated using three donor plasmids containing the Gbait sequences [19]: pGbait-acta1_20 bp-mAG, which possesses short homologous sequences (20 bp each) on both ends of the insert gene fragment (mAG-pA); pGbait-acta1_40 bp-mAG, which possesses short homologous sequences (40 bp each) on both ends of the insert gene; and pGbait-acta1_500 bp-mAG, which possesses longer homologous sequences (472 and 473 bp) on both ends of the insert gene (Fig. 1a). When an integration event occurred precisely into the target site, green fluorescence was observed in the skeletal muscle. As shown in Table 1 and Fig. 1b, 17 of 70 (24.3%) injected embryos expressed green fluorescence in the skeletal muscle following injection of the long homologous plasmid. In contrast, only 2 of 110 (1.9%) and 3 of 64 (4.7%) embryos expressed green fluorescence in skeletal muscle following injection of short homologous plasmids (20_bp and 40_bp, respectively) (Table 1). This suggests that donor plasmids with longer homologous sequences (ca. 500_bp) are more efficiently integrated at the target site of this locus.

**Table 1:** Comparison of integrate efficiency among each of donor plasmids

Length of homology arms	Bait sequences	Survival at 4 dpf	No GFP	GFP+	Integrate efficiency (%)
500 bp	+	70	53	17	24.3
500 bp	-	62	56	6	9.7
40 bp	+	64	61		4.7
20 bp	+	110	108	2	1.9

Integrate efficiency (%) = GFP+ / Survival at 4 dpf

Previous studies have reported that the induction of DSBs on sgRNA target sequences next to homologous sequences in circular donor plasmids using the CRISPR/Cas9 system can enhance targeted integration events [13, 16]. We also evaluated the efficiency of targeted gene integration using donor plasmids containing the long homologous sequences for the *acta1* gene with (pGbait-acta1_500 bp-mAG) or without (pNoBait-acta1_500_bp-mAG) the Gbait sequence containing long homologous sequences of *acta1*. Without the bait sequence, the integration efficiency was reduced to 9.7%, compared to 24.3% with the bait sequence Table 1. This highlights the importance of the bait sequence in efficient targeted gene integration events in medaka Table 1.

To investigate the precision of gene knockin events using the donor plasmid with bait sequences and long homologous sequences (pGbait-acta1_500 bp-mAG), we extracted genomic DNA from five G0 embryos with GFP fluorescence in skeletal muscle (Fig.1b). Each fragment containing an upstream or downstream junction between the genomic sequence and the insert gene was PCR-amplified from the genomic DNA (Additional file 4: Figure S2a) and was sequenced. In all five embryos examined, the donor plasmid was precisely integrated on the target site without any indels (Additional file 4: Figure S2b), indicating that the gene knockin events occurred via HR, as expected.

### Design of bait sequences for targeted gene integration in teleost fish species

To improve the efficiency of targeted gene integration events in medaka and other teleost species, bait sequences with high DSB-inducing activity and without off-target effects must be designed. In this study, we selected 40 sgRNA target sequences with the highest DSB-inducing activity from a dataset from high-throughput screening of mammalian cells [17] as candidates for use as new bait sequences (Additional file 5: Table S2).

To screen bait sequences with fewer off-target effects in a wide range of teleost fish species, we investigated the number of potential off-target sites of 40 candidates in reference genome sequences of medaka and other 11 teleost species (*Danio rerio*, *Gasterosteus aculeatus*, *Oreochromis niloticus*, *Takifugu rubripes*, *Salmo salar*, *Oncorhynchus mykiss*, *Nothobranchius furzeri*, *Astyanax mexicanus*, *Pagrus major*, *Thunnus orientalis*, and *Cynoglossus semilaevis*) (Additional file 1: Table S1). The numbers of genomic sequences with up to 3-bp mismatches in a total of 21 bp of the candidates and a NRG PAM were counted (Additional file 5: Table S2) and the top seven sequences with the fewest potential off-target numbers (#3, #10, #11, #13, #26, #28, and #33) were selected as candidates. We subsequently examined in detail the numbers of mismatch base pairs in all potential off-target sites of these seven candidates and four previously designed bait sequences—Gbait [19] and PITCh gRNAs (the PITCh-gRNA#1-#3) [12]. Ten candidates except the PITCh-gRNA#3 had some potential off-target sites with 2-bp mismatches in the 18-bp target sequence, but none of the 11 candidates had sites with 1 bp or less (Additional file 6: Table S3). We excluded three sequences (#13, #26, the PITCh-gRNA#2) with larger numbers of genomic sites with 2-bp mismatches. Consequently, we included five bait candidates (#3, #10, #11, #28, and #33; hereafter BaitA–E, respectively) and three previously reported sequences (Gbait, PITCh-gRNA#1 and PITCh-gRNA#3) in our evaluation in medaka embryos.

### Comparison of targeted integration efficiencies among bait sequences in medaka

**Fig. 2:**
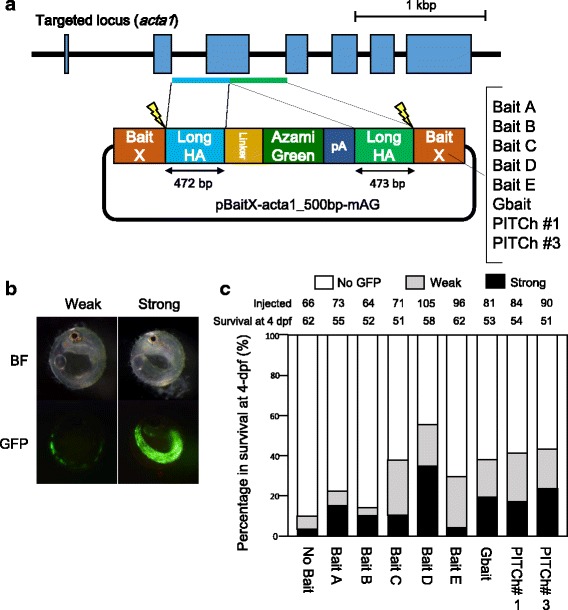
Comparison of the efficiencies of targeted gene integration among bait sequences. (a) Schematic illustration of the genomic targeted locus, *acta1*, and donor plasmids containing each bait sequence. Long homology arms (Long HAs) are shown in boxes with light blue or green. (b) Representative GFP expression in injected embryos at four days post fertilization (dpf). The injected embryos are categorized by GFP expressing area into the following three groups; “No GFP”, “Weak”, or “Strong” with no fluorescence, less than 40% fluorescence, or more than 40% fluorescence of the skeletal muscle, respectively. (c) Evaluation of the efficiencies of targeted gene integration of donor vectors with various bait sequences. The number of injected eggs is shown as “Injected”, and the number of survived embryos at 4-days post fertilization (dpf) among the injected eggs is shown as “Survival at 4 dpf”

We compared the targeted gene integration activity of the five selected bait sequences with those of the previously designed bait sequences, Gbait [19] and PITCh gRNAs (the PITCh-gRNA#1 and PITCh-gRNA#3) [12], which have been reported to exhibit high targeted gene integration activity. A mixture of each donor plasmid with a type of bait sequences, a sgRNA corresponding to each bait sequence, Cas9 RNA, and sgRNA-acta1 was injected into one-cell stage medaka embryos. Expression of green fluorescence in skeletal muscle was observed at 4 days post fertilization (dpf). According to the area expressing green fluorescence, the larvae were categorized into the following three groups: “Strong,” in which green fluorescence was observed in >40% of the area of whole embryonic body; “Weak,” in which green fluorescence was observed in <40% of the area of whole embryonic body; and “No GFP,” in which no fluorescence was detected (Fig. 2b and c).

As shown in Fig. 2c, with all donor plasmids, larvae expressing green fluorescence were observed and all donor plasmids containing bait sequence showed a higher green fluorescence expressing ratio (13.5–55.2%) than those with no bait (without bait sequence) (9.6%). The ratio of larvae with green fluorescence varied and depended on the bait sequences. The highest rate (55.2%) of the larvae with green fluorescence (total larvae with green fluorescence) was observed with BaitD. The ratio of “Strong” area of GFP expression was also highest in BaitD larvae (Fig. 2c). These findings indicate that, of the bait systems tested, the BaitD system most efficiently induces targeted gene integration events. Unfortunately, all of the fish expressing green fluorescence in skeletal muscle died before or soon after hatching; however, the cause of death was unclear.

### Germline transmission of a DNA fragment integrated at the target locus using the BaitD system

**Fig. 3:**
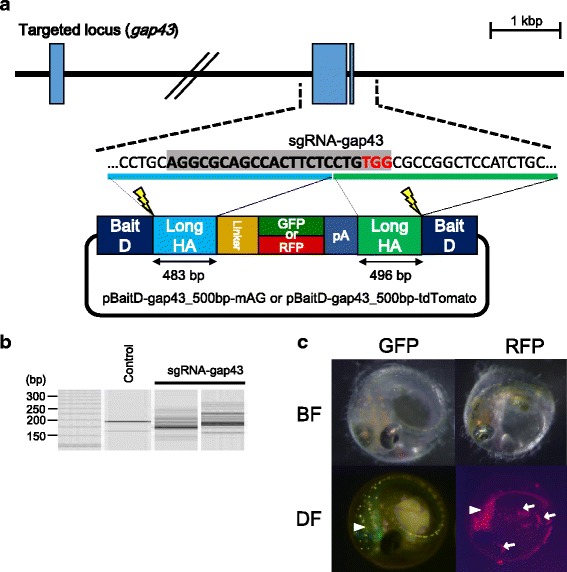
Targeted gene integration of GFP or RFP constructs containing BaitD into the *gap43* locus. (a) Design of sgRNA and homology arms to target the second exon of *gap43* gene (Ensembl transcript number ENSORLT00000015837). The donor plasmids contain two BaitD sequences, upstream and downstream homology arms, and monomeric Azami-Green (mAG; as GFP) or tandem-dimer-Tomato (tdTomato; as RFP) with a N-terminal linker and a SV40 polyA signal (pA). The upstream or downstream homology arms (483 or 496 bp) for the donor plasmids are shown in boxes with light blue or green color, respectively. (b) The genome-editing activity by the sgRNA targeting to the *gap43* locus (sgRNA-gap43), which was investigated using the heteroduplex mobility assay (HMA) of embryos injected with a mixture containing 100 ng/μL of Cas9 mRNA and 50 ng/μL of sgRNA-gap43. Control shows the result from an embryo without injection. (c) GFP and RFP expression in the injected embryos at 4 days post fertilization (dpf). Embryos injected with the donor vectors expressed either GFP or RFP in their central nervous system (CNS). White arrowheads show fluorescence in the CNS, while white arrows show autofluorescence of bacteria on the surface of the egg membrane

**Fig. 4:**
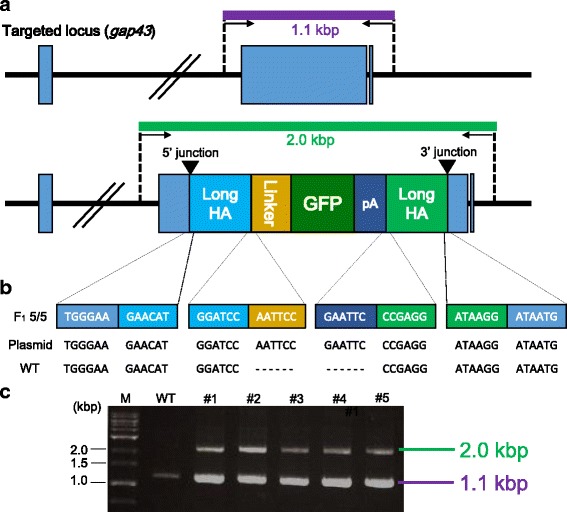
Precise gene integration of a donor vector containing the GFP gene into the *gap43* locus. (a) Schematic illustration of genotyping at the locus. The primer pair (GAP43-for-Seq-Fw and GAP43-for-Seq-Rv) was used for investigating the precise integration into the *gap43* loci. The amplicons with or without the insert sequence are 2.0 kbp or 1.1 kbp, respectively. (b) Sequence analysis of the PCR amplicons (2.0 kbp) containing GFP gene. (Upper): Sequence observed in the F_1_ fish with the inserted gene (#1–#5) (Middle): Sequence in the donor plasmid, pBaitD-gap43_500 bp-mAG (Plasmid). (Lower): Sequence in the wild type fish without the insertion (WT). (c) Electrophoresis image of the PCR products. The shorter PCR product (1.1 kbp) amplified from the intact allele was detected in both a wild type (WT) and five F_1_ fish with the insertion (#1–#5). The longer PCR product (2.0 kbp) was detected only in the F_1_ fish with the insertion (#1–#5)

To investigate whether gene fragments integrated at the target loci using the BaitD system can be transmitted to progeny, we performed a gene knockin experiment targeting another gene, growth associated protein 43 (*gap43*; Ensembl gene number ENSORLG00000015837). The *gap43* transcript is expressed in the central nervous system (CNS) from 4 dpf and contributes to the growth of neuroblasts in medaka [27]. An sgRNA-gap43 with no potential off-target site in the medaka genome was designed on the second exon (Fig. 3a). After confirming the sgRNA possessed high DSB-inducing activity by HMA (Fig. 3b), a mixture of sgRNA-gap43, containing a donor plasmid with two BaitD sequences, a sgRNA for the BaitD sequence, and the Cas9 RNA, was injected into the one-cell stage medaka eggs. Green fluorescence was expressed strongly in the brains of 39 of 320 injected eggs at 4 dpf (Fig. 3c) (Table 2). This fluorescence pattern corresponded to the endogenous expression pattern of the *gap43* gene as reported in a previous study [27]. Twenty-eight of the GFP-expressing embryos were raised to adulthood and mated with wild-type fish. Of the 28 adult fish, two individuals transmitted the insert sequence to the next generation (34.6% and 26.8% germline transmission rate, respectively) (Tables 2 and 3). To investigate whether the knockin events occurred precisely at the target locus, we sequenced the DNA surrounding the inserted fragment after PCR amplification using genomic DNA extracted from five F_1_ embryos with GFP fluorescence in the CNS (Fig. 4a-c). In all five embryos examined, the insert fragment was precisely integrated on the target site without any indels (Fig. 4b), demonstrating that the BaitD system enables effective and precise targeted gene integration in germ cells in medaka.

**Table 2:** Results of microinjection targeting the *gap43* locus with GFP or RFP

	Injected	Survival at 4 dpf	Expressed	Integrate efficiency	Sexually matured	Germ-line transmission rate
GFP	320	158	39	24.7% (39/158)	28	7.1% (2/28)
RFP	103	60	16	26.7% (16/103)	11	9.1% (1/11)

**Table 3:** Germ-line transmition efficiency of F0 individuals harboring GFP or RFP in the gap43 locus

	Collected eggs	Expressed embryos	Transmit efficiency (%)
GFP#1	55	19	34.6
GFP#2	56	15	26.8
RFP#1	58	15	25.9

Transmit efficiency (%) = Expressed embryos / Collected eggs

### PCR genotyping-free selection of double allelic gene edited fish using two different fluorescent colors

To establish a novel genotyping method using two different fluorescent colors, a donor plasmid (pBaitD-gap43_500 bp-tdTomato) containing RFP (*tdTomato* gene) was introduced into fertilized medaka eggs following the same method used for the GFP plasmid. RFP expression in the CNS was observed in 16 of 103 injected embryos and one individual transmitted the insert sequence to the next generation (Table 2).

**Fig. 5:**
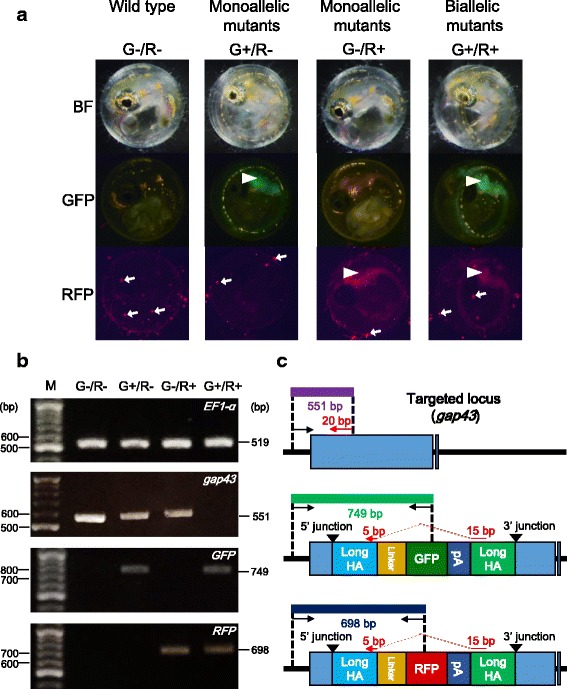
Selection of biallelic mutants using two different colors of fluorescence. (a) Fluorescence of F_2_ embryos derived from mating with each F_1_ monoallelic mutants harboring either the GFP or the RFP gene in the *gap43* locus. Each of the F_2_ embryos were categorized into the following four groups; no fluorescence (G−/R–), green fluorescence (G+/R–), red fluorescence (G−/R+), and both green and red fluorescence (G+/R+). The images were captured at 4 days post fertilization (dpf). White arrowheads show fluorescence in the central nervous system (CNS), while white arrows show autofluorescence of bacteria on the surface of the egg membrane. (b, c) PCR genotyping of the F_2_ embryos derived from mating between the F_1_ monoallelic mutants. (b) The *EF1-α* fragment (519 bp) was used to confirm genomic DNA extraction, and was detected in all samples. The *gap43* fragment (551 bp) was detected from the groups (G−/R–), (G+/R–), and (G−/R+). The GFP fragment (749 bp) was detected from (G+/R–) and (G+/R+) groups, while the RFP fragment (698 bp) was detected from (G−/R+) and (G+/R+) groups. In (G+/R+), no PCR product of *gap43* was detected. (c) Design of PCR primers to detect the targeted gene integration into the *gap43* locus. (Upper): Genomic structure of the intact *gap43* locus. The size of the PCR product amplified using primer pair (GAP43-for-Seq-Fw and GAP43-Rv) is 551 bp. The latter primer is 20 bp in size and is shown as a red arrow. The reverse primer is designed on the boundary between upstream and downstream long homology arms, so that this primer pairs can amplify the intact locus but not the GFP- or RFP-integrated loci. (Middle): Genomic structure of *gap43* locus at which the donor plasmid harboring the GFP gene is integrated. The size of the PCR product amplified using the primer pair (GAP43-for-Seq-Fw and mAG-Rv) is 749 bp. (Lower): Genomic structure of the *gap43* gene at which the donor plasmid harboring the RFP gene is integrated. The size of the PCR product amplified using the primer pair (GAP43-for-Seq-Fw and tdTomato-Rv) is 698 bp

To investigate whether the double allelic gene knockin fish could be selected simply by fluorescence without PCR genotyping, the F_1_ fish harboring the RFP gene in the *gap43* locus was mated with a F_1_ individual harboring the GFP gene in the locus. The resultant F_2_ individuals were divided into the following four groups by color of fluorescence: green fluorescence (G+/R–; *n* = 12), red fluorescence (G−/R+; *n* = 11), both green and red fluorescence (G+/R+; *n* = 13), and no fluorescence (G−/R–; *n* = 11) (Fig. 5a). This distribution indicates that the inserted gene fragments were transmitted in a Mendelian manner. With the genomic DNA extracted from the embryo of each group, PCR analysis was carried out to discriminate among the two inserted gene fragments (GFP and RFP) and an intact allele of the *gap43* gene. As shown in Fig. 5b, in the group “G+/R–”, a GFP allele and an intact allele were detected, indicating that one of the *gap43* alleles was disrupted by the insertion of the GFP gene. Similarly, one of the *gap43* alleles was disrupted by the insertion of the RFP gene in the group “G−/R+”. In the group “G+/R+”, both the GFP and RFP genes were detected in the *gap43* locus, indicating that both alleles were disrupted by the inserted genes. These results indicate that, by mating fish harboring fluorescent reporter genes of different colors in a targeted locus, the genotypes of their progeny (fish without any mutations, monoalleleic mutants, and biallelic mutants) can be determined simply by fluorescent observation at the embryonic stage.

## Discussion

Previous studies have suggested that the length of homologous sequences of donor plasmids is important in determining which DSB repair pathway can be induced by targetable nuclease systems [10]. Targeted gene integration mediated by MMEJ using plasmids with short homologous arms (10–40 bp) has been applied in cultured cells, frogs, mice, and zebrafish, as short arms can be inserted into the donor plasmids easily by PCR or oligonucleotide annealing [11, 12, 14, 28]. Although our results showed that the donor plasmid with 40 bp of homologous arms slightly improved the integration efficiency than that with 20 bp of arms, the efficiencies were much lower than that of the plasmid with longer homology arms (~500 bp each) in medaka (Table 1). These results indicate that donor plasmids with longer homology arms (ca. 500 bp) represent a potentially attractive system for targeted gene integration in medaka. One previous study showed that MMEJ is active during G1 and early S phases when HR is inactive [28]. It has also been reported that some molecules are involved in MMEJ but not in HR [10]. The activity of the DSB repair pathways induced by the nuclease systems may vary among species and across developmental stages. Thus, specifying pathways that are highly active in species and/or developmental stages of interest is needed to establish highly efficient systems for targeted gene integration in any given model system.

Previous studies have reported that simultaneous cleavage of the bait sequences of a donor plasmid and a genomic target site by targetable nucleases can improve the targeted integration efficiency of the plasmid [13, 16]. In our experiments, donor plasmids cleaved by bait systems also showed higher integration efficiencies than that of a donor plasmid with no targeted cleavage (Fig. 2c), which suggests that linearization of donor plasmids using bait systems enhances integration efficiency by HR in medaka.

We additionally designed a new bait sequence BaitD and demonstrated successful targeted gene integration using donor plasmids harboring the bait sequences. Our data indicate that the BaitD system represents a potentially useful system for targeted gene integration with high efficiency in medaka and other fish species, for the following reasons. 1) In an in vivo screening by gene knockin in medaka embryos, the BaitD system showed the highest integration efficiency among eight bait sequences tested (Fig. 2c). 2) Unlike Gbait, the BaitD system can be used in previously established GFP-transgenic strains, as there is no potential target site of the sgRNA for the BaitD in *EGFP* gene. 3) In silico screening in the reference genomes of 12 fish species showed that the BaitD system has less potential off-target effect sites in fish genomes (Additional file 5: Table S2 and Additional file 6: S3).

To date, to identify the genotype of a targeted locus in genome-edited animals, genomic DNA extraction from a tissue sample and the subsequent PCR-based genotyping works have been required. However, these processes are invasive, laborious, and time-consuming. In the present study, we demonstrated a simple method to genotype the genome-edited fish using two different colors of fluorescent protein genes inserted at the target locus (Fig. 5a). Using this method, we were able to determine the genotype of each individual simply by observing the fluorescence; no sacrifice of individuals or labor-intensive processes such as genomic DNA preparation were required. This is especially advantageous for cases in which it is difficult to obtain genomic DNA from living embryos and larvae, as for example in the genotyping of mutants with embryonic lethal phenotypes or in the selection of fish harboring desired mutations at early stages (to reduce breeding costs). Thus, generation of gene knockin strains harboring the different colors of fluorescent protein genes may also represent an effective approach for targeted mutagenesis in medaka and others.

Although some G_0_ fish injected with components to target the *gap43* locus transmitted the genes integrated at the target site to their progenies, the transmission rates were lower than those of other methods in mimicking endogenous gene expression by reporter genes. One example is the homology-independent gene knockin using donor plasmids with a hsp70 promoter and a reporter gene in zebrafish [30]. That study reported that 5.0–10.0% of injected embryos showed broad reporter gene expression and 30.0–40.0% transmitted the gene to next generation; that is, 1.5–4.0% of injected embryos became transgenic founders. In our study, targeted integration of reporter genes into the *gap43* locus resulted in transmission by only 0.6–1.0% of injected embryos to their progeny (Table 2). This lower efficiency may be attributable to our use of promoter-less constructs in the present study. While constructs harboring a promoter can express when integrated in any direction and frame, in-frame integrations into the genomic target site are required for expression of promoter-less constructs. Thus, even if constructs with or without a promoter are integrated at similar efficiencies, the integration efficiency of the promoter-less constructs will be lower than that of constructs with promoter. In another example, bacterial artificial chromosome (BAC)-based transgenes exhibited with high germline transmission rate (~15%) mediated by Tol2 transposon in zebrafish [31]. Although the integration rate of our method is lower than that of the BAC transgenesis, our gene knockin system has the advantage that the transgenes can be precisely integrated into the target site with no positional effects. However, the lower rates of germline transmission in our system indicate the need for further studies to establish methods for improving the efficiency of targeted gene integration in medaka.

Here, we have established an efficient system for targeted gene integration in medaka, which is applicable to the generation of medaka strains with a wide range of genetic modifications. In addition to the gene tagging and knockout by fluorescent reporter genes as shown in this study, this system may enable generation of conditional knockout strains by the insertion of a site-specific recombinase (e.g. LoxP sites for Cre recombinase or FRT sites for Flp) at the genomic targeted position, and for precise introduction of site-specific point mutations in genes of interest [13, 15]. These types of advanced genome editing by HR-mediated targeted gene integration will be useful in accelerating the detailed analysis of gene functions in medaka. Recently, targeted mutagenesis with small indels by the targetable nucleases was also demonstrated in a wide range of fish species in some similar ways to medaka and zebrafish [29–38]. Our findings in improving the efficiency of targeted gene integration may thus be effective for establishing targeted gene integration systems in other fish species as well.

## Conclusions

In this study, we demonstrated targeted gene integration events using CRISPR/Cas9 system and donor plasmids with homologous sequences in medaka. First, we showed that plasmids with longer homology arms (~500 bp each) induced targeted gene integration events more efficiently than those with short homology arms (20 bp and 40 bp). We also found that linearization of the circular donor plasmid by site-specific cleavage with the Cas9 nuclease and the sgRNA targeting to bait sequences increased the efficiency of HR-mediated gene integration in medaka. In addition, a comparison of five newly designed bait sequences and three previously reported sequences revealed that the new bait sequence, BaitD, exhibited the highest efficiency of targeted integration in medaka embryos. Using donor plasmids with longer homology arms and the BaitD sequence, we successfully established gene knockin strains for the *gap43* locus. Taken together, these results open new avenues into the establishment of efficient methods for targeted gene integration using the CRISPR/Cas system in medaka.

## Additional files

Additional file 1: Table S1. Reference genome sequences of teleost species used in this study. (XLSX 9 kb)

Additional file 2: Table S4. Oligonucleotide sequences used in this study. (XLSX 13 kb)

Additional file 3: Figure S1. Selection of sgRNA targeting to the skeletal muscle-specific actin gene (*acta1*) in medaka. (Upper): The design of sgRNAs targeting the *acta1* locus. (Lower): An electrophoresis image shows results of the heteroduplex mobility assay (HMA) in embryos injected with 50 ng/μL of a sgRNA (sgRNA-acta1 #1 or #2) and 100 ng/μL of Cas9 mRNA. Control shows a result from an embryo without injection. (PPTX 76 kb)

Additional file 4: Figure S2. Precise gene integration of a donor vector containing the GFP gene into the *acta1* locus. (a) Schematic illustration of the locus. The primer pair (acta1-for-Seq-Fw and mAG-Rv or mAG-Fw and acta1-for-Seq-Rv) was used for investigating the precise integration into the *acta1* locus. The amplicons containing 5′ junction or 3′ junction are 1.0 kbp, respectively. (b) The sequence analysis of the PCR amplicons (1.0 kbp) containing 5′ or 3′ junction. (Upper): The sequence observed in the G_0_ fish with the inserted gene (#1–#5). (Middle): The sequence in the donor plasmid, pBaitD-acta1_500 bp-mAG (Plasmid). (Lower): The sequence in the wild type fish without the insertion (WT). (PPTX 41 kb)

Additional file 5: Table S2. The numbers of potential off-target sequences in reference genomes of teleost fish species. Numbers of genomic sites with up to 3 bp mismatches in the total 21 bp sequences are shown. (XLSX 11 kb)

Additional file 6: Table S3. Potential off-target sites of 7 candidates of bait sequence that selected in the first screening and previously reported bait sequences. Potential off-target sites are defined as genomic sequence harboring up to 2 bp mismatches in the total 18 bp sequences and a NGG PAM. (XLSX 11 kb)
