# Correction to: An efficient system for homology-dependent targeted gene integration in medaka (*Oryzias latipes*)

**DOI:** 10.1186/s40851-019-0139-x

**Published:** 2019-07-08

**Authors:** Yu Murakami, Satoshi Ansai, Akari Yonemura, Masato Kinoshita

**Affiliations:** 1 Division of Applied Bioscience, Graduate School of Agriculture, Kyoto University, Kitashirakawa-Oiwake-cho, Sakyo-ku, Kyoto, 606-8502 Japan; 2 Present address: Division of Ecological Genetics, Department of Population Genetics, National Institute of Genetics, Yata 1111, Mishima, Shizuoka, 411-8540 Japan

**Correction to: Zoological Lett**

**https://doi.org/10.1186/s40851-017-0071-x**

Please note that there are two errors present in the tables of the published article [1].

Firstly, the value ‘3’ is missing from the 5th row of the ‘GFP+’ column of Table 1.

**Table 1:** Comparison of integrate efficiency among each of donor plasmids

Length of homology arms	Bait sequences	Survival at 4 dpf	No GFP	GFP+	Integrate efficiency (%)
500 bp	+	70	53	17	24.3
500 bp	–	62	56	6	9.7
40 bp	+	64	61	3	4.7
20 bp	+	110	108	2	1.9

Secondly, the gene sequence given for ‘Candidate #28’ in Additional file 6: Table S3 is incorrect. The gene sequence should be ‘TCTTCGGCCTAGACTGCGAGG’.

Please find the corrected tables below for reference:

## Additional file

Additional file 6: **Table S3.** Potential off-target sites of 7 candidates of bait sequence that selected in the first screening and previously reported bait sequences. Potential off-target sites are defined as genomic sequence harboring up to 2 bp mismatches in the total 18 bp sequences and a NGG PAM. (XLSX 10 kb)
