# Supplementary material for: An efficient system for homology-dependent targeted gene integration in medaka (Oryzias latipes)
Source: Zoological Lett. 2017 Jul 6;3:10. doi: 10.1186/s40851-017-0071-x (PMC5500998; doi:10.1186/s40851-017-0071-x)
Supplement: Supplementary file 3 — Selection of sgRNA targeting to the skeletal muscle-specific actin gene (acta1) in medaka. (Upper): The design of sgRNAs targeting the acta1 locus. (Lower): An electrophoresis image shows results of the heteroduplex mobility assay (HMA) in embryos injected with 50 ng/μL of a sgRNA (sgRNA-acta1 #1 or #2) and 100 ng/μL of Cas9 mRNA. Control shows a result from an embryo without injection. (PPTX 76 kb) [file 40851_2017_71_MOESM3_ESM.pptx]

## Slide 1
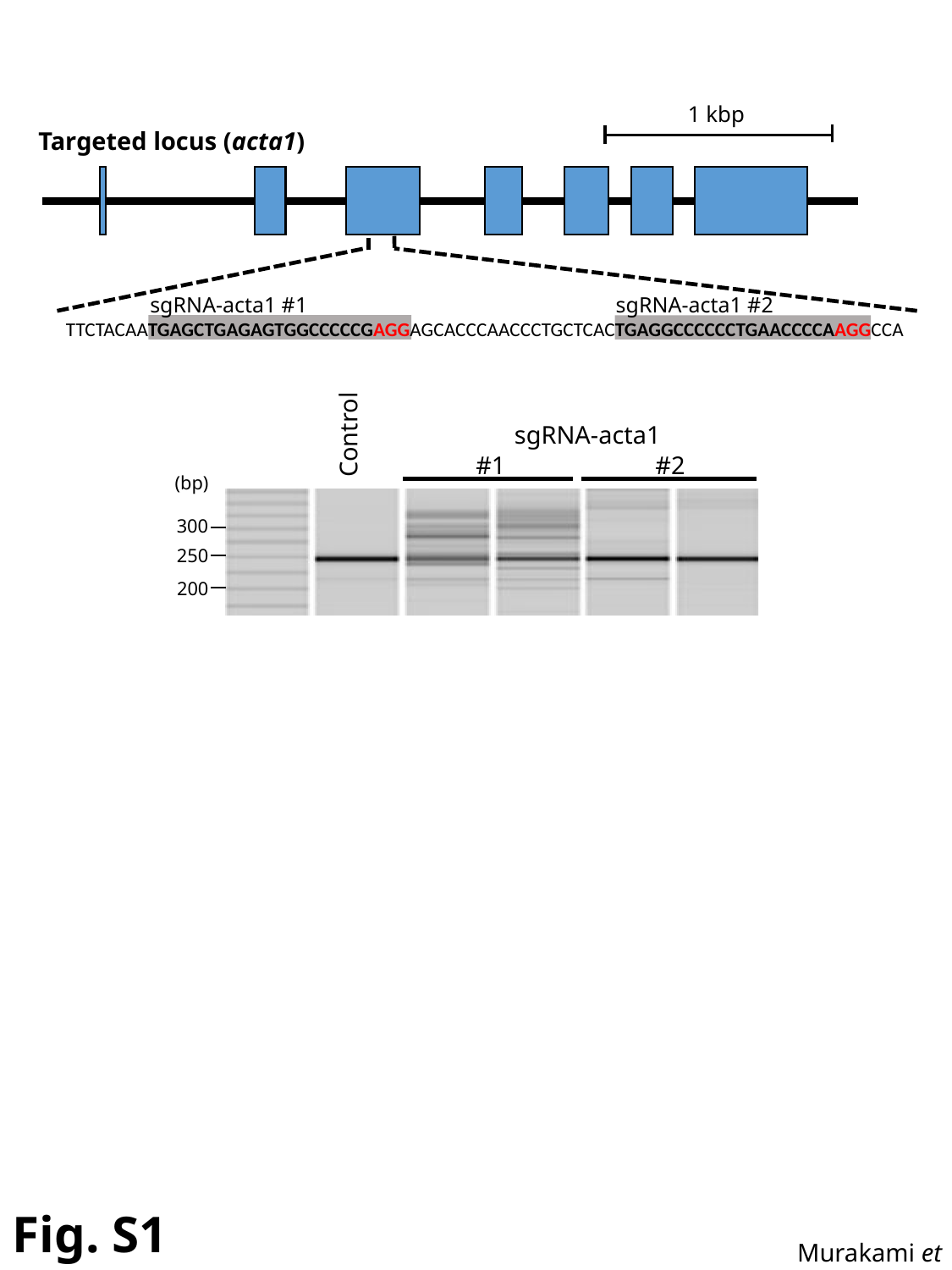

1 kbp
Targeted locus (acta1)
sgRNA-acta1 #1
sgRNA-acta1 #2
TTCTACAATGAGCTGAGAGTGGCCCCCGAGGAGCACCCAACCCTGCTCACTGAGGCCCCCCTGAACCCCAAGGCCA
Control
sgRNA-acta1
#1
#2
(bp)
300
250
200
Fig. S1
Murakami et al.
