# Supplementary material for: An efficient system for homology-dependent targeted gene integration in medaka (Oryzias latipes)
Source: Zoological Lett. 2017 Jul 6;3:10. doi: 10.1186/s40851-017-0071-x (PMC5500998; doi:10.1186/s40851-017-0071-x)
Supplement: Supplementary file 4 — Precise gene integration of a donor vector containing the GFP gene into the acta1 locus. (a) Schematic illustration of the locus. The primer pair (acta1-for-Seq-Fw and mAG-Rv or mAG-Fw and acta1-for-Seq-Rv) was used for investigating the precise integration into the acta1 locus. The amplicons containing 5′ junction or 3′ junction are 1.0 kbp, respectively. (b) The sequence analysis of the PCR amplicons (1.0 kbp) containing 5′ or 3′ junction. (Upper): The sequence observed in the G0 fish with the inserted gene (#1–#5). (Middle): The sequence in the donor plasmid, pBaitD-acta1_500 bp-mAG (Plasmid). (Lower): The sequence in the wild type fish without the insertion (WT). (PPTX 41 kb) [file 40851_2017_71_MOESM4_ESM.pptx]

## Slide 1
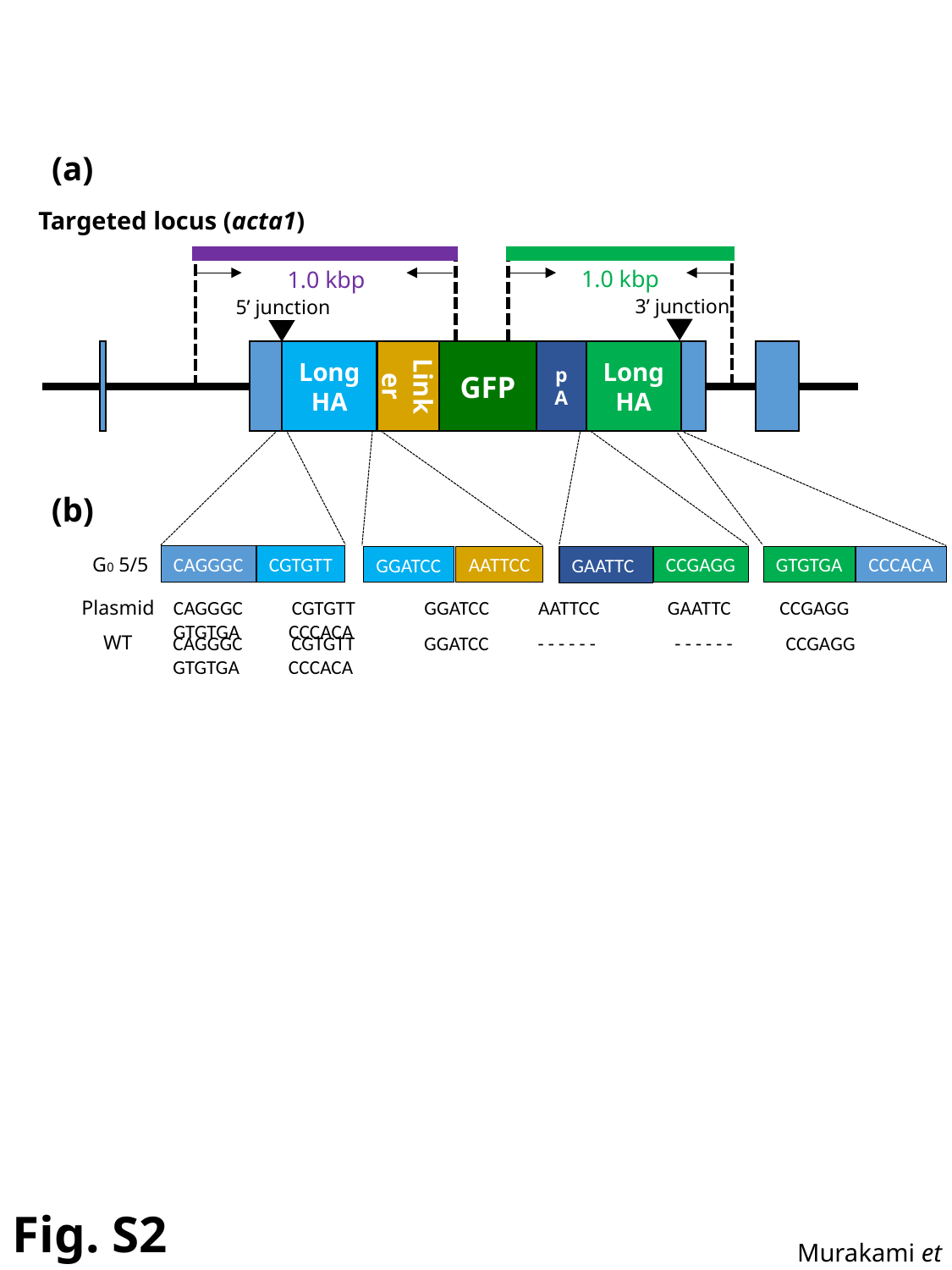

(a)
Targeted locus (acta1)
1.0 kbp
1.0 kbp
3’ junction
5’ junction
Long
HA
Long
HA
Linker
GFP
pA
(b)
CAGGGC
CGTGTT
G0 5/5
AATTCC
CCCACA
GTGTGA
GAATTC
CCGAGG
GGATCC
Plasmid
CAGGGC　　CGTGTT　　 GGATCC　　AATTCC 　 　 GAATTC　　CCGAGG　 　GTGTGA　　CCCACA
WT
CAGGGC　　CGTGTT　　 GGATCC　　- - - - - -　 　 - - - - - - 　　CCGAGG　 　GTGTGA　　CCCACA
Fig. S2
Murakami et al.
